# Persistence of Yellow fever virus outside the Amazon Basin, causing epidemics in Southeast Brazil, from 2016 to 2018

**DOI:** 10.1371/journal.pntd.0006538

**Published:** 2018-06-04

**Authors:** Izabela Maurício de Rezende, Lívia Sacchetto, Érica Munhoz de Mello, Pedro Augusto Alves, Felipe Campos de Melo Iani, Talita Émile Ribeiro Adelino, Myrian Morato Duarte, Ana Luísa Furtado Cury, André Felipe Leal Bernardes, Tayrine Araújo Santos, Leonardo Soares Pereira, Maria Rita Teixeira Dutra, Dario Brock Ramalho, Benoit de Thoisy, Erna Geessien Kroon, Giliane de Souza Trindade, Betânia Paiva Drumond

**Affiliations:** 1 Laboratório de Vírus, Departamento de Microbiologia, Universidade Federal de Minas Gerais, Belo Horizonte, Minas Gerais, Brazil; 2 Centro de Controle de Zoonoses da Prefeitura de Belo Horizonte, Belo Horizonte, Minas Gerais, Brazil; 3 Laboratório de Imunologia de Doenças Virais, Instituto René Rachou- Fundação Oswaldo Cruz, Belo Horizonte, Minas Gerais, Brazil; 4 Serviço de Virologia e Riquetsioses, Fundação Ezequiel Dias- LACEN/MG, Belo Horizonte, Minas Gerais, Brazil; 5 Hospital Eduardo de Menezes, Fundação Hospitalar do Estado de Minas Gerais, Belo Horizonte, Minas Gerais, Brazil; 6 Laboratoire des Interaction Virus-Hôtes, Institut Pasteur, Cayenne, French Guiana; Institute for Disease Modeling, UNITED STATES

## Abstract

**Background:**

Yellow fever (YF) is endemic in the Brazilian Amazon Basin, and sporadic outbreaks take place outside the endemic area in Brazil. Since 2016, YF epidemics have been occurring in Southeast Brazil, with more than 1,900 human cases and more than 1,600 epizooties of non-human primates (NHPs) reported until April 2018. Previous studies have demonstrated that Yellow fever virus (YFV) causing outbreaks in 2017 formed a monophyletic group.

**Methodology/Principal findings:**

Aiming to decipher the origin of the YFV responsible for the recent epidemics, we obtained nucleotide sequences of YFV detected in humans (n = 6) and NHPs (n = 10) from Minas Gerais state during 2017–2018. Next, we performed evolutionary analyses and discussed the results in the light of epidemiological records (official numbers of YFV cases at each Brazilian Federative unit, reported by the Brazilian Ministry of Health). Nucleotide sequences of YFV from Southeast Brazil from 2016 to 2018 were highly conserved and formed a monophyletic lineage (BR-YFV_2016/18) within the genotype South America I. Different clusters were observed within lineage BR-YFV_2016/18, one containing the majority of isolates (from humans and NHPs), indicating the sylvatic transmission of YFV. We also detected a cluster characterized by two synapomorphies (amino acid substitutions) that contained YFV only associated with NHP what should be further investigated. The topology of lineage BR-YFV_2016/18 was congruent with epidemiological and temporal patterns of the ongoing epidemic. YFV isolates detected in 2016, in São Paulo state were located in the most basal position of the lineage, followed by the isolates from Minas Gerais and Espírito Santo obtained in 2017 and 2018. The most recent common ancestor of the lineage BR-YFV_2016/18 dated to 2015 (95% credible intervals = 2014–2016), in a period that was coincident with the reemergence of YFV in the Midwest region of Brazil.

**Conclusions:**

The results demonstrated a single introduction of YFV in the Southeast region and the silent viral circulation before the onset of the outbreaks in 2016. Evolutionary analyses combined with epidemiological records supported the idea that BR-YFV_2016/18 was probably introduced from the Midwest into the Southeast region, possibly in São Paulo state. The persistence of YFV in the Southeast region, causing epidemics from 2016 to 2018, suggests that this region presents suitable ecological and climatic conditions for YFV maintenance during the epidemic and interepidemic seasons. This fact poses risks for the establishing of YF enzootic cycles and epidemics, outside the Amazon Basin in Brazil. YF surveillance and studies of viral dynamics deserve particular attention, especially in Midwest, Southeast and neighbor regions which are the main areas historically associated with YF outbreaks outside the Amazon Basin. YFV persistence in Southeast Brazil should be carefully considered in the context of public health, especially for public health decision-makers and researchers.

## Author summary

Yellow fever (YF) is endemic in the Brazilian Amazon Basin, but sporadic outbreaks may take place outside this region. At the end of 2016, YF epidemics have been occurring in Southeast Brazil, with thousands of human cases (more than 1,900) and deaths of non-human primates (NHPs) reported so far. To better understand the origin and dynamics of YFV associated with these epidemics, we studied part of the viral genome obtained from humans and NHPs, in 2016 to 2018. All YFV grouped within a single group, indicating a single event of virus introduction in Southeast Brazil. Epidemiological data combined with our results suggested that the virus might have been introduced from the Midwest into the Southeast region, one year before the first outbreak was detected (2016). The persistence of YFV in the Southeast, causing epidemics from 2016 to 2018 indicates that this region presents suitable conditions for YFV maintenance, during the epidemic and non-epidemic periods. This fact may represent a risk for YFV establishment in Southeast Brazil. This information should be carefully considered by public health decision-makers and researchers in Brazil.

## Introduction

*Yellow fever virus* (YFV) (family *Flaviviridae*, genus *Flavivirus*) is the etiologic agent of yellow fever (YF). In Brazil, the urban cycle has not occured since 1942, but sylvatic yellow fever (SYF) occurs in the Amazon Basin (S1A Fig) in an enzootic cycle involving non-human primates (NHPs) and sylvatic vectors such as *Haemagogus* sp. and *Sabethes* sp. [1,2]. Outside the Amazon Basin, SYF reemerges with an irregular annual periodicity, but with a seasonality pattern, with the majority of cases occurring between December and May [2]. It is believed that YFV is disseminated to other areas outside the Amazon Basin by the movement of infected and viremic people or by the illegal traffic of infected NHPs [1].

In 1999, the epidemiological pattern of SYF in Brazil has changed, as the majority of human cases have been reported in states from Midwest, Southeast and South region located (S1B Fig) outside the Amazon Basin (S1B Fig), mainly during the wet season [3]. In 2000–2001, during a YF outbreak in the states of Minas Gerais (MG) and São Paulo (SP), a total of 98 human cases were registered, and epizootics were also reported in the states of Bahia, Paraná and Rio Grande do Sul. Another outbreak outside the Amazon Basin took place in 2008–2009, with 57 human cases reported in Southeast (MG and SP), Northeast (Bahia and Rio Grande do Norte), and South (Rio Grande do Sul) regions. Epizooties were also registered in the states of MG, SP, Rio Grande do Sul and Paraná [4,5].

At the end of 2016, a large epidemic of YF started in Southeast Brazil. Until July 2017, 1,412 epizootics, 777 YF human cases, and 261 deaths were registered, mostly in the Southeast region [6]. During the interepidemic season, YF cases were reported in NHPs but not in humans [7]. At December 2017, human YF outbreaks reemerged in Southeast Brazil. A total of 1,127 human cases and 331 deaths have been confirmed (from July 2017 to April 2018) by the Brazilian Ministry of Health, mostly in the Southeast region of the country [8]. The SYF outbreaks started in 2016 in MG state which presented low average vaccination coverage (57.26%), at that time. A total of 32.7% of municipalities presented vaccination coverage ≤50%; while 64% of municipalities had vaccination coverage between 50% and 95%, and only 3.3% of municipalities presented vaccination coverage equal or above 95% (S1C Fig) [9]. In MG, from December 2016 to April 2018, 942 human cases and 317 deaths have been confirmed [7,10].

Previous studies of YFV, from the states of Rio de Janeiro and Espírito Santo have suggested that the sylvatic transmission during the outbreak caused by a monophyletic group of YFV subdivided into two subclusters [11,12], with an ancestor estimated to exist in 2016 [11,13]. These studies suggested that YFV dissemination occurred from Venezuela to the Southeast region of Brazil, through intermediate viral migration steps involving North and Midwest regions [11,13].

To better understand the origin and dynamics of recent YFV epidemics in Brazil, we performed evolutionary analysis of YFV detected from human and NHPs from the Southeast region. The results were combined with epidemiological data, bringing some light on the ongoing YF epidemics.

## Methods

### Biological samples

Non-human primates carcasses from nine municipalities (S1C Fig) were received during YF epizootics from January to February 2017, in MG state. Among the carcasses there were individuals from genera *Alouatta*, *Callithrix* and *Callicebus* (S1 Table). The NHP carcasses were collected in different rural areas of MG, where epizootics were reported in 2017 and 2018, and sent to our laboratory. Liver fragments were collected under biosafety conditions and kept at -70°C until RNA extraction.

Sera from patients were received for YF diagnosis in a reference diagnosis laboratory linked to the Brazilian Health Department (LACEN/MG—Laboratório Central de Saúde Pública de Minas Gerais, located at Fundação Ezequiel Dias, MG) or from patients admitted at Hospital Eduardo de Menezes, Belo Horizonte, MG. These sera were from six patients living in five municipalities of MG (S1C Fig) and were collected during the outbreaks in January 2017 (n = 3) and January 2018 (n = 3). Five of the participants were men (from 22 up to 55 years old), and one participant was a 47 years old woman. All these participants lived in municipalities where YF outbreaks occurred in 2017 or 2018 [7,10]. From four participants, we had information about occupation (driver, rural workers, and civil construction worker), area where they lived (rural area), outcome of disease (death), and none of them reported history of travelling previously (15 days) to the onset of the disease (S2 Table).

### Ethics statement

The study was authorized by Minas Gerais state Health Department and approved by Ethics Committee on Human Research of Instituto René Rachou (license CAAE 65814417.0.0000.5091). The Minas Gerais state Health Department and the Ethics Committee on Human Research of Instituto René Rachou approved the analysis of biological samples of patients without the informed consent from each one, as the samples were collected and received for YFV molecular diagnosis and analysis, in different parts of the state. We managed to obtain the written informed consent from four patients admitted at Hospital Eduardo de Menezes. The study was also authorized by the Ethics Committee on Animal Research of Universidade Federal de Minas Gerais (license 98/2017).

### Viral multiplication in cell culture

Sera (20 μL) from three patients from 2017 were inoculated in cell culture tubes (Sarstedt, Australia) with *Aedes albopictus* C6/36 cell monolayers (American Type Culture Collection—CRL-1660). The cells were propagated and maintained in Leibovit**´**z L-15 medium (Gibco, USA) supplemented with 5% fetal bovine serum (Cultilab, Brazil). The cells were incubated at 28°C, for 10 days. After this period, the supernatant was harvested, collected and kept at -70°C until RNA extraction.

### YFV molecular investigation

For each NHP (n = 10), fragments of 30 mg of the liver were used in total RNA extraction, using RNeasy Mini Kit (Qiagen, USA). From human samples, total RNA was extracted using 140 μL of serum (n = 3, sera collected in 2018) or infected cell supernatant (n = 3, sera collected in 2017), using QIAmp Viral RNA Mini Kit (Qiagen, USA). YFV RNA investigation was performed by real-time PCR preceded by reverse transcription (RT-qPCR), using GoTaq Probe 1-Step RT-qPCR System (Promega) and primers and probe described by Domingo and colleagues (2012) [14]. Using specific primers, targeting CprM and envelope regions of the YFV genome [15], partial sequences were amplified and sequenced by dideoxy-method on an ABI3130 platform (Applied Biosystems). Raw data were analyzed and final contigs were assembled using SeqTrace [16].

### Phylogenetic and evolutionary analyses

Based on the availability of BR-YFV sequences in databases, in February 2018, we worked with two datasets, containing sequences from CprM/envelope region and used these datasets for phylogenetic and evolutionary analyses. The first dataset included 60 YFV nucleotide (nt) sequences, spanning 1,038 nt (from the nt 125 to 1,162 of ES504 (KY885000) sequence), from South America and African genotypes. The first dataset included 22 sequences of BR-YFV obtained from 2016 to 2018 plus 13 sequences of BR-YFV from previous years (BR-YFV sequences: n = 35) (S3 Table). To include a greater number of sequences from Latin America and Brazil, we performed the analyses with a second dataset, using a different part of CprM/envelope region (n = 125 sequences, 651 nt, nt 644 to 1,294 of ES504 (KY885000) sequence). This second dataset included 18 sequences of BR-YFV obtained in 2017 and 2018 plus 57 sequences of BR-YFV from previous years (BR-YFV sequences: n = 75) (S4 Table). The nt sequences were aligned with MAFFT multiple sequence alignment program [17] and the alignments were used to perform evolutionary analyses. Phylogenetic trees using the Maximum likelihood method implemented in PhyML 3.0 [18] were reconstructed. The nucleotide substitution model TN+G was selected using SMS [19] and for tree search the SPR branch-swapping algorithm was used followed by the approximate likelihood-ratio test (aLRT) to access the support of branches. Analyses to check temporal signal of sequences were performed using TempEst v.1.5.1 [20], previously to analyses to infer the time of the most recent common ancestor (MRCA) of YFV causing the outbreaks in 2000–01, 2008–09, and the current one (2016–2018). Analyses were performed using BEAST package v.1.8.4 [21] with Markov Chain Monte Carlo algorithms. Input files for BEAST v.1.8.4 were created with BEAUTi v.1.8.2 [21]. The calibration point was the year in which each virus was obtained. Runs were performed using the different demographic coalescent models (parametric and non-parametric) under strict or relaxed (uncorrelated lognormal) molecular clock and using the estimated rate of 5×10^−4^ substitutions per site [13]. The best model was selected comparing the marginal likelihood estimations, using path sampling (PS) and stepping-stone sampling (SS) methods [22]. The estimates were performed nucleotide substitution model HKY, with gamma distribution (four categories), under the relaxed molecular clock and Bayesian skyline demographic model. One hundred million chains were run, the first 10 million steps were discarded, convergence of parameters was verified with Tracer v.1.5.0 [23], and uncertainties were addressed as the 95% Bayesian credible intervals (BCI). The trees were sampled at every 10,000 steps and then summarized in a maximum clade credibility tree using TreeAnotator v.1.8.2 [24]. Chains were run for three independent times and data were combined using LogCombiner v.1.7.4 [25]. The final tree was visualized in FigTree v.1.4.3 [26].

Epidemiological data of retrospective cases of YFV in humans and NHPs (date and place where the cases were reported) were collected from official bulletins of the Brazilian Ministry of Health and records of SINAN (Sistema National de Agravos de Notificação), a platfform for registration of notifiable diseases in Brazil [5,27]. This information was used to discuss the results obtained here.

## Results

All human (n = 6) and NHP (n = 10) samples tested positive by RT-qPCR, confirming the infection by YFV. YFV nt sequences (n = 16) spanning 1,259 nt from capsid to envelope region were obtained (Genbank accession numbers: MG838679—MG838688, MH001693—MH001695 and MH015342-MH015344). Sequences of YFV collected in 2017 and 2018 were highly conserved (99.8–100% of nt identity). In some of the predicted amino acid sequences of BR-YFV obtained from NHPs, two mutations were observed in the envelope gene sequence, which resulted in two amino acid substitution in positions 15 (V to L), and 16 (H to Y).

**Fig 1:**
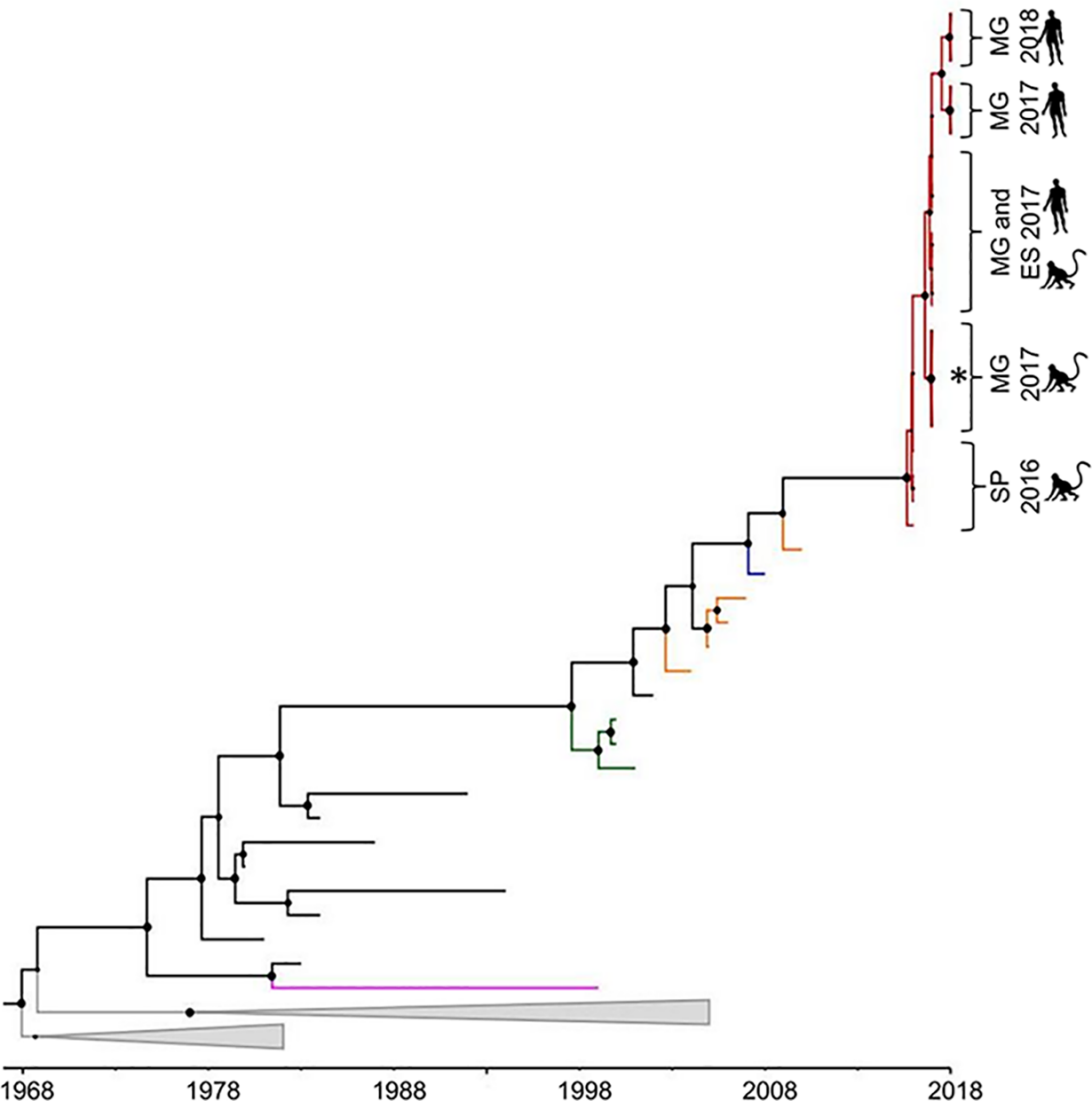
Bayesian phylogenetic analyses of Yellow fever virus. The maximum clade credibility tree inferred using 60 Yellow fever virus sequences (1,038 nt) from South America is shown. The posterior probability values are represented by circles drawn in scale in the nodes. Clades containing Brazilian Yellow fever virus from outbreaks 2000–01, 2008–09 and the current (2016–2018) are shown in green, blue and red, respectively. Terminal branches in orange, pink and black represent sequences of Yellow fever virus from Venezuela, Bolivia, and Brazil, respectively. South African genotypes are collapsed in grey. Horizontal branch lengths are drawn to a scale of years. The tree was reconstructed using the nucleotide substitution model HKY with gamma distribution (four categories), under the relaxed molecular clock and Bayesian skyline demographic model. ES: Espírito Santo state, MG: Minas Gerais state and SP: São Paulo state. The asterisk (*) denotes the cluster of Yellow fever virus obtained from non-human primates which had two amino acid substitutions characterized as synapomorphies. Analyses were performed using programs from BEAST package v.1.8.4 [21], BEAUTi v.1.8.2 [21], Tracer v.1.5.0 [23], TreeAnotator v.1.8.2 [24] and FigTree v.1.4.3 [26].

Phylogenetic analyses based on Maximum likelihood (S1 Fig) and Bayesian methods (Fig 1) demonstrated that BR-YFVs from 2016 to 2018, formed a monophyletic lineage, called BR-YFV_2016/18 (supported by a posterior probability (PP) equal to 1). This lineage clustered within South America genotype I. Using the second dataset, BR-YFVs from 2017 and 2018, also formed a monophyletic cluster highly supported (PP = 1) (S3 Fig). BR-YFVs within the lineage BR-YFV_2016–18 were closely related to YFV from Venezuela (Fig 1 and S3 Fig). BR-YFVs from outbreaks that took place in 2000–01 (Midwest, and Southeast regions) and 2008–09 (Midwest, South, and Southeast regions) formed distinct lineages (PP>0.91), called here lineages BR-YFV_2000/01 and BR-YFV_2008/09, respectively) (Fig 1 and S3 Fig). The monophyletic lineage BR-YFV_2016/18 was subdivided into different clusters (Fig 1) with YFV from 2016 in a basal position within the lineage. One cluster contained BR-YFV (obtained from five NHPs, from genera *Alouatta* sp., *Callithrix* sp., and *Callicebus* sp.), presenting two synapomorphies regarding the amino acid substitutions in positions 15 and 16 of deduced envelope protein. Another cluster contained BR-YFVs, from 2017 and 2018, obtained from NHPs (*Alouatta* sp., *Callithrix* sp., and *Callicebus* sp.) and humans (Fig 1).

Using dataset 1 containing 22 sequences of BR-YFVs from 2016 to 2018, the MRCA of lineage BR-YFV_2016/18 was estimated at July 2015 (95% Bayesian credible intervals (95%BCI) = from July 2014 to January 2016). The MRCA of BR-YFVs from 2017 and 2018 was estimated to exist in May 2016 (95%BCI = from July 2015 to January 2017). For estimation of MRCA of older BR-YFV lineages, we used the second dataset that contained more YFV sequences from previous outbreaks. The lineage BR-YFV_2008/09 had the MRCA dated at 2007 (95%BCI = from 2005 to 2008), and the MRCA of lineage BR-YFV_2000/01 was estimated to exist in the middle of 1998 (95%BCI = from 1997 to 1999).

## Discussion

Here we aimed to decipher the origin of the YFV responsible for more than 593 deaths in Brazil, from 2016 to April 2018. For this purpose, we performed molecular analyses of BR-YFV associated with human epidemics and epizootics in NHPs. Nucleotide sequences of 16 BR-YFVs were highly conserved and formed a monophyletic lineage called BR-YFV_2016/18, confirming a single event of introduction of YFV in the Southeast region, causing the ongoing outbreak. After the introduction in the Southeast region, this lineage has been locally evolving, giving rise to different subgroups. Previous findings, based on analysis of BR-YFVs obtained in 2017, from states of Rio de Janeiro, Minas Gerais and Espírito Santo [11,12], support this conclusion. Within the lineage BR-YFV_2016/18, we observed one cluster composed by YFVs from human and different species of NHPs, indicating the sylvatic transmission during the outbreaks, as previously demonstrated [12]. Another cluster which presented two synapomorphies (two amino acid substitutions in the deduced envelope protein), grouped YFV strains associated with NHPs. These synapomorphies were not associated with any NHP genera as they were observed in specimens obtained from *Alouatta* sp., *Callithrix* sp., and *Callicebus* sp. Moreira-Soto and colleagues (2018) [12] also observed the presence of a basal YFV group (from 2017) associated with NHPs. These data may indicate the existence of a cluster within lineage BR-YFV_2016/18 only associated with epizootics in NHPs, but a greater number of strains from human and NHPs should be analyzed to test this hypothesis.

BR-YFVs obtained from 2016 to 2018 from SP, MG and Espírito Santo states clustered within lineage BR-YFV_2016/18, sharing a common ancestor estimated to exist in the middle of 2015. Moreover, this lineage has persisted from this time up to 2018, infecting humans and NHPs in the Southeast region. This lineage was closely related to strains from Venezuela, and in fact, some studies have proposed that YFV causing the recent outbreak was probably introduced into Brazil, from Venezuela [11–13]. After introduction into Brazil, the virus would have reached the Southeast region, possibly through intermediate migration steps through North and Midwest regions [11,13]. However, there are very few nt sequences of BR-YFV from previous years (obtained at different times and geographic regions) making the phylogeographic analyses to test this hypothesis difficult. Combining our results with epidemiological records (date and place where previous cases of human YF and epizootics took place in Brazil), we obtained some insights about the origin of YFV outbreaks outside the Amazon Basin, in Brazil.

The MRCA of lineage BR-YFV_2016/18 was estimated to exist in a period (middle of 2015, 95% BCI = from July 2014 to January 2016) that was coincident to YFV reemergence and circulation in the Midwest region. In July 2014, SYF reemerged in the Midwest region (state of Goiás) and, based on temporal and spatial epidemiological data, the virus disseminated and reached the Southeast region (SP state), in 2016 [2,28]. In the Midwest region, at least six human cases had the most probable location of infection in the states of Goiás (Midwest) and Tocantins (North), in areas with intense touristic activity [2] which could have contributed to the viral dissemination to other areas, as the Southeast region. From July 2014 to December 2016, 21 YF epizootics were confirmed in Southeast and two human cases were confirmed in SP state [2,28].

In a similar way, the MRCAs of BR-YFV lineages that caused outbreaks in 2008–2009 and 2000–2001 were estimated to exist 1 to 2 years before their detection. The 2008–2009 outbreak took place in Midwest, South and Southeast regions of Brazil from 2008 onwards. However, the MRCA of these viruses was estimated to exist at 2007 (95%BCI = 2005–2008). This period coincides with the time that human cases were reported in the Midwest region in 2006, in the state of Mato Grosso (n = 1) and, shortly after, in 2007, in the state of Goiás (n = 2) [29]. Based on epidemiological records, YFV was possibly introduced into the Midwest region (18 confirmed epizootics) and disseminated to Southeast and South regions (191 confirmed epizootics) [4].

The MRCA of lineages BR-YFV_2016/18 and BR-YFV_2008/09 were dated in a period when SYF was reported in the Midwest region. These facts support the hypothesis that YFV causing those outbreaks (2016–18, and 2008–09) would have been originated in the Midwest region. In fact, Brazilian health authorities already postulated that the detection of YFV in the Midwest region should be fully addressed with the intensification of viral surveillance [2], given the emergence of SYF related to this region. Indeed, Mir and colleagues (2017) [13] confirmed the viral dissemination from the Midwest region to Southeast and South regions related to the 2000–2001 outbreak, by phylogeographical analyses. Thus, phylogeographical analyses [13], epidemiological records combined with the evolutionary data presented here, support the idea that the Midwest region may be a hot spot for YFV emerging outside the Amazon Basin.

The gap (1 to 2 years) between the date of MRCAs of BR-YFV lineages (BR-YFV_2000/01, BR-YFV_2008/09, and BR-YFV_2016/18) and the detection of SYV demonstrate that YFV is maintained in a silent circulation outside the Amazon Basin, for a period before its detection. Here, we showed the introduction of YFV, followed by its persistence in the Southeast region, for more than 3 years. Recent outbreaks in Brazil also confirmed the intense YFV circulation during epidemic periods [29] and non-epidemics periods [2,30] in Southeast and Midwest regions [2,30]. These data suggest that areas outside the Amazon Basin present suitable ecological and climatic conditions for YFV maintenance, even during interepidemic periods. In fact, based on the records of YF infection and vaccination coverage, Shearer and colleagues (2018) [31] identified the Southeast coast of Brazil (states of Bahia, MG, SP, Espírito Santo, and Rio de Janeiro) as an area of high receptivity to YFV transmission.

In previous outbreaks, the numbers of human and NHP cases were considerably smaller than the current epidemic [29]. The cause of the explosive character of the epidemics/epizootics in MG and neighbor states is still unclear, but the silent circulation of YFV might have contributed to the geographic dissemination until the epidemic seasons started (2016–2017 and 2017–2018). These facts added to the vaccination coverage [32] which was very low in some regions of MG (including the regions where the outbreaks took place), until 2016 might have contributed to the magnitude of this ongoing YF epidemic. Moreover, genome sequences have revealed mutations leading to amino acid substitutions constituting synapomorphies of lineage BR-YFV_2016/18, in the capsid and in non-structural proteins [11,12]. However, this is still an open debate, and further studies should be conducted to better understand the dynamics of the current YF epidemics, in the light of virological, ecological, evolutionary, immunological, and epidemiological aspects.

Finally, the results support a single introduction of YFV in the Southeast region, approximately in 2015, and viral persistence and local evolution until 2018. This is particularly worrisome as the Southeast region concentrates 44.7% of the Brazilian population and it is classified as an area of high receptivity to YF transmission. Yellow fever surveillance, including human, sentinel animals and entomological surveillance, coupled with studies on viral dynamics deserve particular attention in Brazil, mainly in Southeast, Midwest and adjacent regions.

## Supporting information

S1 Fig Historical data of yellow fever in Brazil. **(A)** Geopolitical map of Brazil and the Amazon Basin. The Amazon Basin, where sylvatic yellow fever is endemic, is highlighted. Brazilian geopolitical regions are colored as follows: North region in blue, Northeast region in dark orange, Southeast region in green, South region in purple, and Midwest region in red. Federal District and States are represented by initials, as follows: AC: Acre; AL: Alagoas; AP: Amapá; AM: Amazonas; BA: Bahia; CE: Ceará; DF: Distrito Federal: ES: Espírito Santo; GO: Goiás; MA: Maranhão; MT: Mato Grosso; MS: Mato Grosso do Sul; MG: Minas Gerais; PA: Pará; PB: Paraíba; PR: Paraná; PE: Pernambuco; PI: Piauí; RR: Roraima; RO: Rondônia; RJ: Rio de Janeiro; RN: Rio Grande do Norte; RS: Rio Grande do Sul; SC: Santa Catarina; SP: São Paulo; SE: Sergipe; and TO: Tocantins. The map was done using QGIS v.2.18.16 [33] and information from Instituto Brasileiro de Geografia e Estatística [34] and Ministério do Meio Ambiente [35]. **(B)** Number of sylvatic yellow fever human cases, from 2001 to February 2018, according to the regions of Brazil. The y-axis is shown in logarithmic scale and indicates the number of cases per year. The Brazilian regions and the bars representing the numbers of yellow fever human cases are colored accordingly. *data from May 2016 to July 2017 [6] and ** data from July 2017 to February 2018 [36]. Data regarding the number of yellow fever cases were obtained from Sistema de Informação de Agravos de Notificação (SINAN) and official bulletins from Ministry of Health, Brazil. **(C)**. Map of Minas Gerais State showing the vaccination coverage per municipatilty in 2016 (data available at http://www.saude.mg.gov.br/febreamarela, accessed at April 20th, 2018). Red: municipalities with vaccination coverage ≤50%; yellow: municipalities with vaccination coverage between 50% and 95%, and green: municipalities with vaccination coverage equal or above 95%. The municipalities where human or non-human samples were collected are indicated by dots and respective names.
(TIF)

S2 Fig Phylogenetic analyses of Yellow fever virus (maximum likelihood). The tree was inferred using 60 Yellow fever virus sequences (1,038 nt) from South America, and the Maximum likelihood method implemented in PhyML 3.0. The nucleotide substitution model TN+G was used, and the approximate likelihood-ratio test (aLRT) to access the support of branches (values are represented by circles drawn into scale at nodes). Yellow fever virus strains from South American Genotypes are shown in green, and the Brazilian yellow fever virus from outbreaks 2016–2018 are collapsed in a triangle. Horizontal branch lengths are drawn to a scale of nucleotide substituion per site according to the scale.
(TIF)

S3 Fig Phylogenetic analyses of Yellow fever virus (based on dataset 2). The maximum clade credibility tree inferred using 125 Yellow fever virus sequences (651 nt) from South America is shown. The posterior probabilities are represented by circles drawn in scale in the nodes. Clades containing Brazilian yellow fever virus from outbreaks 2000–01, 2008–09 and the current (2016–2018) are shown in green, blue and red, respectively. Terminal branches in orange represent sequences of Yellow fever virus from Venezuela, pink from Colombia, purple from Trinidad and Tobago, light green from Panama, light blue from Ecuador and black from Brazil. Sequences from South America, genotpe II and South African genotype are collapsed in grey. Horizontal branch lengths are drawn to a scale of years. The tree was reconstructed using the nucleotide substitution model HKY with gamma distribution (four categories), under the relaxed molecular clock and Bayesian skyline demographic model. The asterisk (*) denotes the cluster of *Yellow fever virus* obtained from non-human primates which had two amino acid substitutions characterized as synapomorphies. ES: Espírito Santo state, MG: Minas Gerais state. Analyses were performed using programs from BEAST package v.1.8.4 [21], BEAUTi v.1.8.2 [21], Tracer v.1.5.0 [23], TreeAnotator v.1.8.2 [24] and FigTree v.1.4.3 [26]
(TIF)

S1 Table Information regarding non-human primate carcasses. Jan: January. Feb: February. ^a^All municipalities are located in Minas Gerais state, Southeast Brazil. Location of each municipality can be observed in S1C Fig.
(DOC)

S2 Table Information regarding patients. M: Male. F: Female. NA: not available. ^a^All municipalities are located in Minas Gerais State, Southeast Brazil. ^b^Years. Jan: January. Location of each municipality can be observed in S1C Fig.
(DOC)

S3 Table Information of Yellow fever virus sequences included into dataset 1. YFV: Yellow fever virus. ID: identification. This dataset included 60 YFV nucleotide (nt) sequences, spanning 1,038 nt (from the nucleotide 125 up to 1,162 of ES504 (KY885000) sequence) from South American and African genotypes. The dataset included 22 sequences of BR-YFV obtained from 2016 up to 2018 plus 13 sequences of BR-YFV from previous years (BR-YFV sequences: n = 35).
(DOC)

S4 Table Information of Yellow fever virus sequences included into dataset 2. YFV: Yellow fever virus. ID: identification. This dataset contained 125 sequences spanning 651 nt (from the nucleotide 644 up to 1,294 of ES504 (KY885000) sequence). This dataset included 18 sequences of BR-YFV obtained in 2017 and 2018 plus 57 sequences of BR-YFV from previous years (BR-YFV sequences: n = 75).
(DOC)
