# Supplementary figures and images for: Persistence of Yellow fever virus outside the Amazon Basin, causing epidemics in Southeast Brazil, from 2016 to 2018
Source: PLoS Negl Trop Dis. 2018 Jun 4;12(6):e0006538. doi: 10.1371/journal.pntd.0006538 (PMC6002110; doi:10.1371/journal.pntd.0006538)

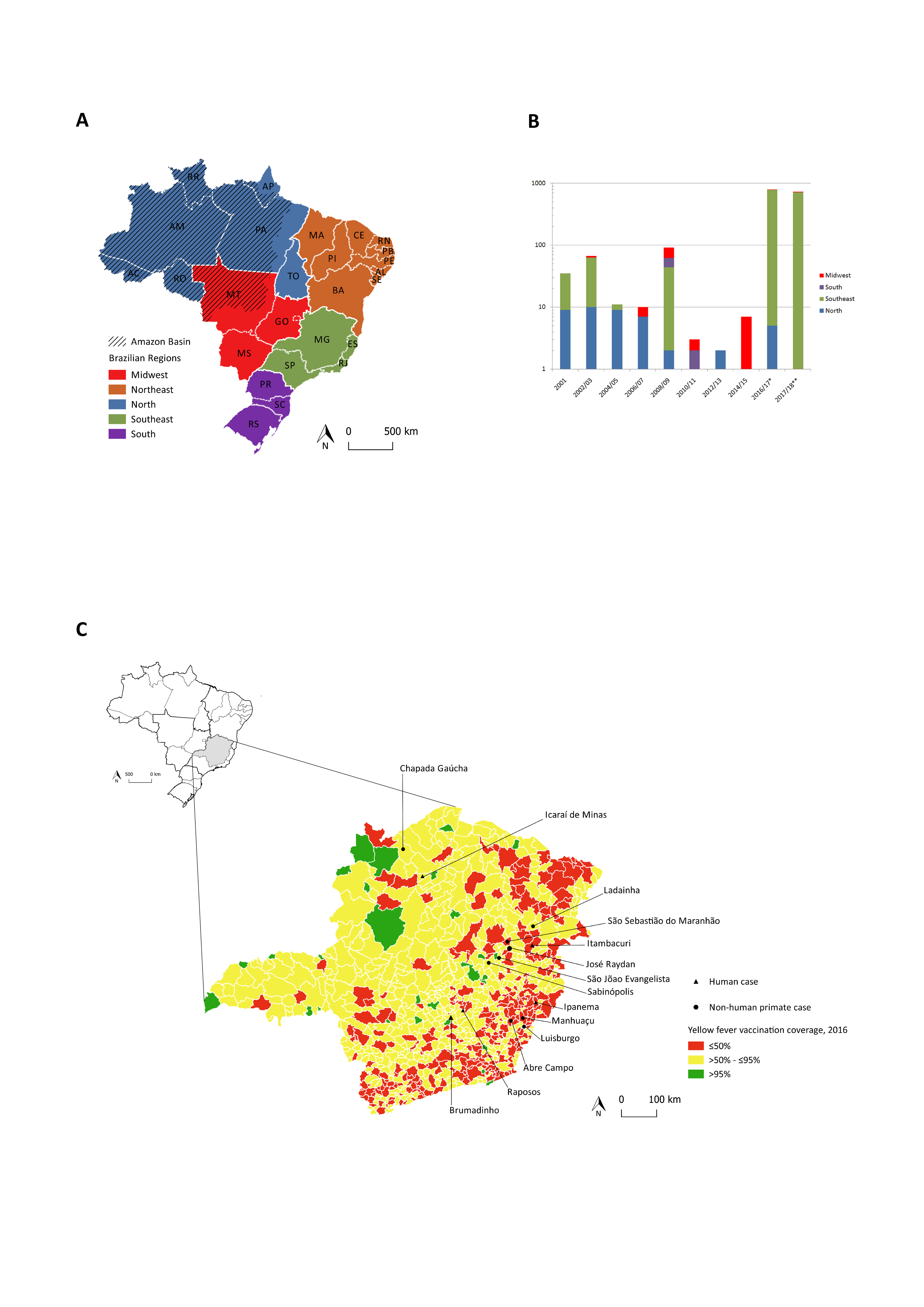

Supplement: S1 Fig — (A) Geopolitical map of Brazil and the Amazon Basin. The Amazon Basin, where sylvatic yellow fever is endemic, is highlighted. Brazilian geopolitical regions are colored as follows: North region in blue, Northeast region in dark orange, Southeast region in green, South region in purple, and Midwest region in red. Federal District and States are represented by initials, as follows: AC: Acre; AL: Alagoas; AP: Amapá; AM: Amazonas; BA: Bahia; CE: Ceará; DF: Distrito Federal: ES: Espírito Santo; GO: Goiás; MA: Maranhão; MT: Mato Grosso; MS: Mato Grosso do Sul; MG: Minas Gerais; PA: Pará; PB: Paraíba; PR: Paraná; PE: Pernambuco; PI: Piauí; RR: Roraima; RO: Rondônia; RJ: Rio de Janeiro; RN: Rio Grande do Norte; RS: Rio Grande do Sul; SC: Santa Catarina; SP: São Paulo; SE: Sergipe; and TO: Tocantins. The map was done using QGIS v.2.18.16 [33] and information from Instituto Brasileiro de Geografia e Estatística [34] and Ministério do Meio Ambiente [35]. (B) Number of sylvatic yellow fever human cases, from 2001 to February 2018, according to the regions of Brazil. The y-axis is shown in logarithmic scale and indicates the number of cases per year. The Brazilian regions and the bars representing the numbers of yellow fever human cases are colored accordingly. *data from May 2016 to July 2017 [6] and ** data from July 2017 to February 2018 [36]. Data regarding the number of yellow fever cases were obtained from Sistema de Informação de Agravos de Notificação (SINAN) and official bulletins from Ministry of Health, Brazil. (C). Map of Minas Gerais State showing the vaccination coverage per municipatilty in 2016 (data available at http://www.saude.mg.gov.br/febreamarela, accessed at April 20th, 2018). Red: municipalities with vaccination coverage ≤50%; yellow: municipalities with vaccination coverage between 50% and 95%, and green: municipalities with vaccination coverage equal or above 95%. The municipalities where human or non-human samples were collected are indicated by dots [file pntd.0006538.s001.tif]

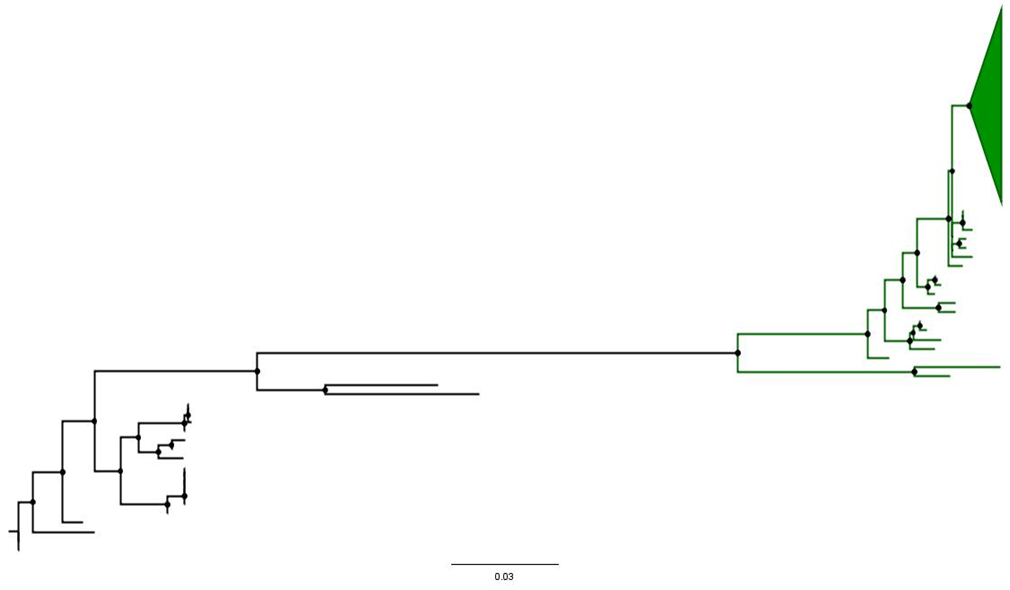

Supplement: S2 Fig — The tree was inferred using 60 Yellow fever virus sequences (1,038 nt) from South America, and the Maximum likelihood method implemented in PhyML 3.0. The nucleotide substitution model TN+G was used, and the approximate likelihood-ratio test (aLRT) to access the support of branches (values are represented by circles drawn into scale at nodes). Yellow fever virus strains from South American Genotypes are shown in green, and the Brazilian yellow fever virus from outbreaks 2016–2018 are collapsed in a triangle. Horizontal branch lengths are drawn to a scale of nucleotide substituion per site according to the scale. (TIF) [file pntd.0006538.s002.tif]

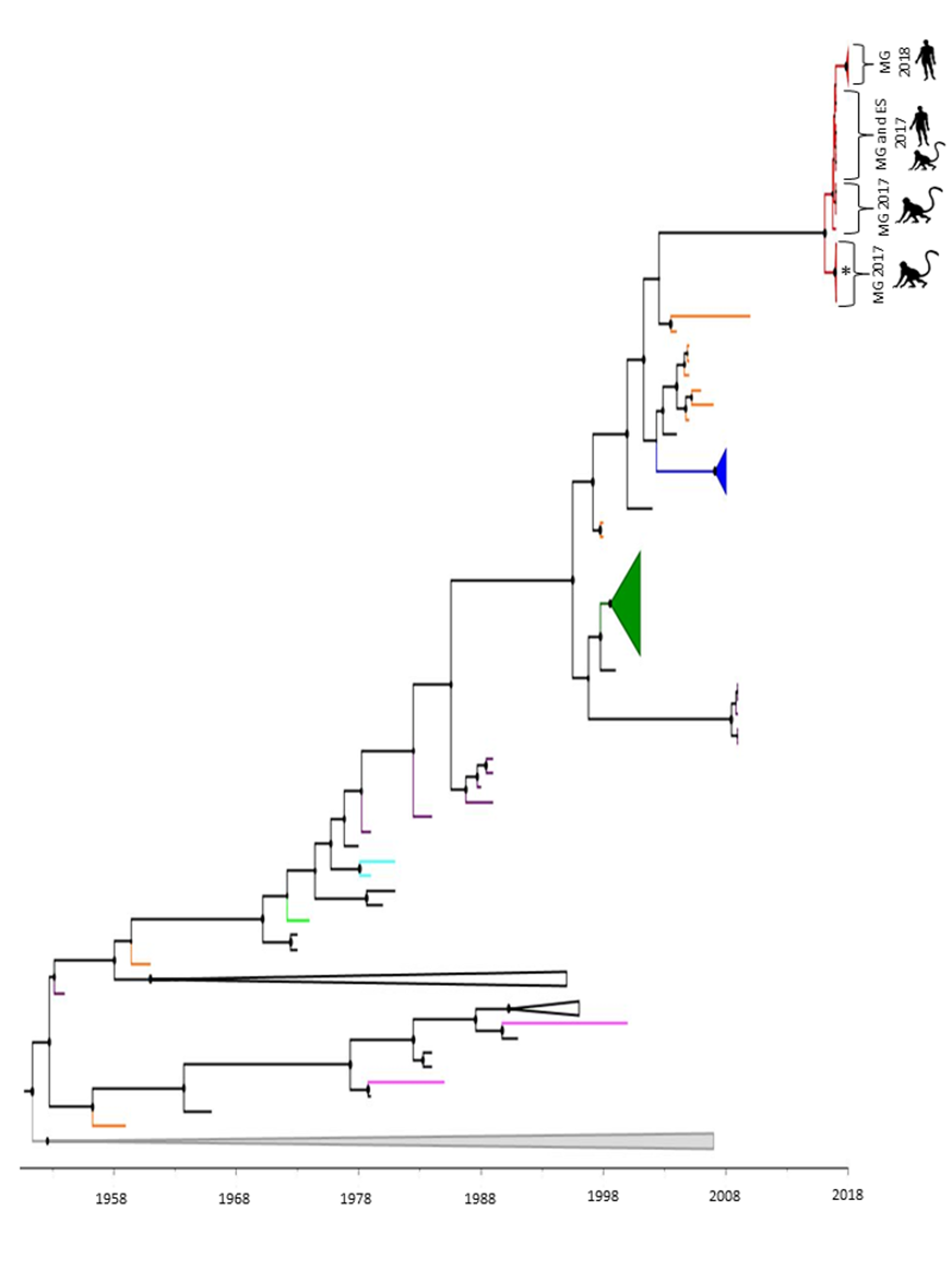

Supplement: S3 Fig — The maximum clade credibility tree inferred using 125 Yellow fever virus sequences (651 nt) from South America is shown. The posterior probabilities are represented by circles drawn in scale in the nodes. Clades containing Brazilian yellow fever virus from outbreaks 2000–01, 2008–09 and the current (2016–2018) are shown in green, blue and red, respectively. Terminal branches in orange represent sequences of Yellow fever virus from Venezuela, pink from Colombia, purple from Trinidad and Tobago, light green from Panama, light blue from Ecuador and black from Brazil. Sequences from South America, genotpe II and South African genotype are collapsed in grey. Horizontal branch lengths are drawn to a scale of years. The tree was reconstructed using the nucleotide substitution model HKY with gamma distribution (four categories), under the relaxed molecular clock and Bayesian skyline demographic model. The asterisk (*) denotes the cluster of Yellow fever virus obtained from non-human primates which had two amino acid substitutions characterized as synapomorphies. ES: Espírito Santo state, MG: Minas Gerais state. Analyses were performed using programs from BEAST package v.1.8.4 [21], BEAUTi v.1.8.2 [21], Tracer v.1.5.0 [23], TreeAnotator v.1.8.2 [24] and FigTree v.1.4.3 [26] (TIF) [file pntd.0006538.s003.tif]
