# Supplementary material for: Persistence of Yellow fever virus outside the Amazon Basin, causing epidemics in Southeast Brazil, from 2016 to 2018
Source: PLoS Negl Trop Dis. 2018 Jun 4;12(6):e0006538. doi: 10.1371/journal.pntd.0006538 (PMC6002110; doi:10.1371/journal.pntd.0006538)
Supplement: S1 Table — Jan: January. Feb: February. aAll municipalities are located in Minas Gerais state, Southeast Brazil. Location of each municipality can be observed in S1C Fig. (DOC) [file pntd.0006538.s004.doc]

**S1 Table. Information regarding non-human primate carcasses.**

| **Samples** | **Date** | **Genera** | **Area** | **Municipality^a^** | **Vaccination coverage in 2016** |
| --- | --- | --- | --- | --- | --- |
| YFV_NHP01_BR_MG_2017 | Jan/2017 | *Callithrix geoffroyi* | Rural | Sabinópolis | 93.83 |
| YFV_NHP03_BR_MG_2017 | Jan/2017 | *Alouatta* cf*. guariba* | Rural | José Raydan | 68.53 |
| YFV_NHP05_BR_MG_2017 | Jan/2017 | *Callithrix geoffroyi* | Rural | Ladainha | 55.06 |
| YFV_NHP07_BR_MG_2017 | Jan/2017 | *Callithrix geoffroyi* | Rural | São Sebastião do Maranhão | 15.30 |
| YFV_NHP09_BR_MG_2017 | Jan/2017 | *Callithrix geoffroyi* | Rural | São João Evangelista | 53.63 |
| YFV_NHP10_BR_MG_2017 | Jan/2017 | *Callicebus* sp. | Rural | Manhuaçu | 45.98 |
| YFV_NHP12_BR_MG_2017 | Jan/2017 | *Callicebus* sp. | Rural | Luisburgo | 97.06 |
| YFV_NHP15_BR_MG_2017 | Jan/2017 | *Alouatta* cf. *guariba* | Rural | Chapada Gaúcha | 82.64 |
| YFV_NHP88_BR_MG_2017 | Feb/2017 | *Callithrix geoffroyi* | Rural | José Raydan | 68.53 |
| YFV_NHP96_BR_MG_2017 | Feb/2017 | *Alouatta* cf. *guariba* | Rural | Abre Campo | 32.83 |

Jan: January. Feb: February. ^a^All municipalities are located in Minas Gerais state, Southeast Brazil. Location of each municipality can be observed in S1 Fig C.
