# Supplementary material for: Persistence of Yellow fever virus outside the Amazon Basin, causing epidemics in Southeast Brazil, from 2016 to 2018
Source: PLoS Negl Trop Dis. 2018 Jun 4;12(6):e0006538. doi: 10.1371/journal.pntd.0006538 (PMC6002110; doi:10.1371/journal.pntd.0006538)
Supplement: S2 Table — M: Male. F: Female. NA: not available. aAll municipalities are located in Minas Gerais State, Southeast Brazil. bYears. Jan: January. Location of each municipality can be observed in S1C Fig. (DOC) [file pntd.0006538.s005.doc]

**S2 Table. Information regarding patients.**

| **Samples** | **Date** | **Outcome** | **Municipality^a^** | **Age^b^** | **Sex** | **Occupation** | **Vaccination coverage in 2016** |
| --- | --- | --- | --- | --- | --- | --- | --- |
| YFV_HS_HEM298_BR_MG_2018 | Jan/2018 | death | Raposos | 30 | M | driver | 52.73 |
| YFV_HS_HEM295_BR_MG_2018 | Jan/2018 | death | Brumadinho | 54 | M | rural worker | 64.44 |
| YFV_HS_HEM306_BR_MG_2018 | Jan/2018 | death | Brumadinho | 40 | M | civil construction worker | 64.44 |
| YFV_HS_SVR581_BR_MG_2017 | Jan/2017 | death | Ipanema | 22 | M | rural worker | 37.21 |
| YFV_HS_SVR267_BR_MG_2017 | Jan/2017 | NA | Itambacuri | 47 | F | NA | 39.91 |
| YFV_HS_SVR371_BR_MG_2017 | Jan/2017 | NA | Icaraí de Minas | 55 | M | NA | 72.22 |

M: male. F: female. NA: not available. ^a^All municipalities are located in Minas Gerais state, Southeast Brazil. ^b^Years. Jan: January. Feb: February.

Location of each municipality can be observed in S1 Fig C.
