# Supplementary material for: Persistence of Yellow fever virus outside the Amazon Basin, causing epidemics in Southeast Brazil, from 2016 to 2018
Source: PLoS Negl Trop Dis. 2018 Jun 4;12(6):e0006538. doi: 10.1371/journal.pntd.0006538 (PMC6002110; doi:10.1371/journal.pntd.0006538)
Supplement: S3 Table — YFV: Yellow fever virus. ID: identification. This dataset included 60 YFV nucleotide (nt) sequences, spanning 1,038 nt (from the nucleotide 125 up to 1,162 of ES504 (KY885000) sequence) from South American and African genotypes. The dataset included 22 sequences of BR-YFV obtained from 2016 up to 2018 plus 13 sequences of BR-YFV from previous years (BR-YFV sequences: n = 35). (DOC) [file pntd.0006538.s006.doc]

**S3 Table. Information of Yellow fever virus sequences included into dataset 1.**

| **YFV strain** | **Origin** | **Date** | **GenBank ID** |
| --- | --- | --- | --- |
| YFV_HS_SVR371_BR_MG_2017 | Brazil | 2017 | MH015233 |
| YFV_HS_SVR581_BR_MG_2017 | Brazil | 2017 | MH015342- |
| YFV_HS_SVR267_BR_MG_2017 | Brazil | 2017 | MH015343 |
| YFV_HS_HEM295_BR_MG_2018 | Brazil | 2018 | MH001694 |
| YFV_HS_HEM306_BR_MG_2018 | Brazil | 2018 | MH001695 |
| YFV_HS_HEM298_BR_MG_2018 | Brazil | 2018 | MH001693 |
| YFV_NHP01_BR_MG_2017 | Brazil | 2017 | MG838679 |
| YFV_NHP03_BR_MG_2017 | Brazil | 2017 | MG838680 |
| YFV_NHP05_BR_MG_2017 | Brazil | 2017 | MG838681 |
| YFV_NHP07_BR_MG_2017 | Brazil | 2017 | MG838682 |
| YFV_NHP09_BR_MG_2017 | Brazil | 2017 | MG838683 |
| YFV_NHP10_BR_MG_2017 | Brazil | 2017 | MG838684 |
| YFV_NHP12_BR_MG_2017 | Brazil | 2017 | MG838685 |
| YFV_NHP15_BR_MG_2017 | Brazil | 2017 | MG838686 |
| YFV_NHP88_BR_MG_2017 | Brazil | 2017 | MG838687 |
| YFV_NHP96_BR_MG_2017 | Brazil | 2017 | MG838688 |
| ES-504 | Brazil | 2017 | KY885000.1 |
| ES-505 | Brazil | 2017 | KY885001.1 |
| JabSPM04 | Brazil | 2016 | MF443187.1 |
| JabSPM03 | Brazil | 2016 | MF443186.1 |
| JabSPM02 | Brazil | 2016 | MF443185.1 |
| JabSPM01 | Brazil | 2016 | MF443184.1 |
| BeAR513008 | Brazil | 1992 | JF912185.1 |
| BeH413820 | Brazil | 1983 | JF912181.1 |
| BeH423602 | Brazil | 1984 | JF912183.1 |
| BeH526722 | Brazil | 1994 | JF912186.1 |
| BeAR646536 | Brazil | 2001 | JF912189.1 |
| BeH622493 | Brazil | 2000 | JF912188.1 |
| BeH422973 | Brazil | 1984 | JF912182.1 |
| BeAR378600 | Brazil | 1980 | JF912179.1 |
| BeH463676 | Brazil | 1987 | JF912184.1 |
| BeH622205 | Brazil | 2000 | JF912187.1 |
| BeH394880 | Brazil | 1981 | JF912180.1 |
| BeH655417 | Brazil | 2002 | JF912190.1 |
| BeAn754036(PR4408) | Brazil | 2008 | KY861728.1 |
| 88/1999 | Bolivia | 1999 | KF907504.1 |
| 2A | Venezuela | 2004 | KM388817.1 |
| 10A | Venezuela | 2010 | KM388816.1 |
| 8A | Venezuela | 2006 | KM388818.1 |
| 9A | Venezuela | 2007 | KM388815.1 |
| 6A | Venezuela | 2005 | KM388814.1 |
| ArD181439 | Senegal | 2005 | JX898881.1 |
| ArD181564 | Senegal | 2005 | JX898880.1 |
| ArD181676 | Senegal | 2005 | JX898879.1 |
| ArD181250 | Senegal | 2005 | JX898878.1 |
| ArD181464 | Senegal | 2005 | JX898877.1 |
| ArD156468 | Senegal | 2001 | JX898876.1 |
| ArD149815 | Senegal | 2000 | JX898875.1 |
| ArD149194 | Senegal | 2000 | JX898874.1 |
| ArD149214 | Senegal | 2000 | JX898873.1 |
| ArD114972 | Senegal | 1995 | JX898872.1 |
| ArD114896 | Senegal | 1995 | JX898871.1 |
| ArD121040 | Senegal | 1996 | JX898870.1 |
| DakArAmt7 | Coted' Ivoire | 1973 | JX898869.1 |
| HD117294 | Senegal | 1995 | JX898868.1 |
| Gambia2001 | Gambia | 2001 | AY572535.1 |
| 85-82H | Ivory Coast | 1982 | U54798.1 |
| YF-AVD2791-93F/04 | Spain | 2004 | DQ118157.1 |
| IvoryCoast1999 | Coted' Ivoire | 1999 | AY603338.1 |
| Trinidad79A_788379 | Trinidad and Tobago | 1979 | AF094612.1 |

YFV: Yellow fever virus. ID: identification. This dataset included 60 YFV nucleotide (nt) sequences, spanning 1,038 nt (from the nucleotide 125 up to 1,162 of ES504 (KY885000) sequence) from South American and African genotypes. The dataset included 22 sequences of BR-YFV obtained from 2016 up to 2018 plus 13 sequences of BR-YFV from previous years (BR-YFV sequences: n=35).
