# Supplementary material for: Persistence of Yellow fever virus outside the Amazon Basin, causing epidemics in Southeast Brazil, from 2016 to 2018
Source: PLoS Negl Trop Dis. 2018 Jun 4;12(6):e0006538. doi: 10.1371/journal.pntd.0006538 (PMC6002110; doi:10.1371/journal.pntd.0006538)
Supplement: S4 Table — YFV: Yellow fever virus. ID: identification. This dataset contained 125 sequences spanning 651 nt (from the nucleotide 644 up to 1,294 of ES504 (KY885000) sequence). This dataset included 18 sequences of BR-YFV obtained in 2017 and 2018 plus 57 sequences of BR-YFV from previous years (BR-YFV sequences: n = 75). (DOC) [file pntd.0006538.s007.doc]

**S4 Table. Information of Yellow fever virus sequences included into dataset 2.**

|  | **YFV strain** | | **Origin** | | **Year** | | **GenBank ID** | |
| --- | --- | --- | --- | --- | --- | --- | --- | --- |
| YFV_HS_SVR371_BR_MG_2017 | | Brazil | | 2017 | | MH015233 | |  |
| YFV_HS_SVR581_BR_MG_2017 | | Brazil | | 2017 | | MH015342- | |  |
| YFV_HS_SVR267_BR_MG_2017 | | Brazil | | 2017 | | MH015343 | |  |
| YFV_HS_HEM295_BR_MG_2018 | | Brazil | | 2018 | | MH001694 | |  |
| YFV_HS_HEM306_BR_MG_2018 | | Brazil | | 2018 | | MH001695 | |  |
| YFV_HS_HEM298_BR_MG_2018 | | Brazil | | 2018 | | MH001693 | |  |
| YFV_NHP01_BR_MG_2017 | | Brazil | | 2017 | | MG838679 | |  |
| YFV_NHP03_BR_MG_2017 | | Brazil | | 2017 | | MG838680 | |  |
| YFV_NHP05_BR_MG_2017 | | Brazil | | 2017 | | MG838681 | |  |
| YFV_NHP07_BR_MG_2017 | | Brazil | | 2017 | | MG838682 | |  |
| YFV_NHP09_BR_MG_2017 | | Brazil | | 2017 | | MG838683 | |  |
| YFV_NHP10_BR_MG_2017 | | Brazil | | 2017 | | MG838684 | |  |
| YFV_NHP12_BR_MG_2017 | | Brazil | | 2017 | | MG838685 | |  |
| YFV_NHP15_BR_MG_2017 | | Brazil | | 2017 | | MG838686 | |  |
| YFV_NHP88_BR_MG_2017 | | Brazil | | 2017 | | MG838687 | |  |
| YFV_NHP96_BR_MG_2017 | | Brazil | | 2017 | | MG838688 | |  |
|  | ES-504 | | Brazil | | 2017 | | KY885000.1 | |
|  | ES-505 | | Brazil | | 2017 | | KY885001.1 | |
|  | BeAn754036(PR4408) | | Brazil | | 2008 | | KY861728.1 | |
|  | BeH622205 | | Brazil | | 2000 | | HM582849.1 | |
|  | BeH35010 | | Brazil | | 1995 | | AY540471.1 | |
|  | BeAn142027 | | Brazil | | 1968 | | AY540448.1 | |
|  | SPH188002 | | Brazil | | 2000 | | FJ875515.1 | |
|  | SPH188057 | | Brazil | | 2000 | | FJ875516.1 | |
|  | SPAn288184 | | Brazil | | 2008 | | FJ875519.1 | |
|  | AY437135 | | Brazil | | 2001 | | AY437135.1 | |
|  | BeH111 | | Brazil | | 1954 | | AY540437.1 | |
|  | BeAN131 | | Brazil | | 1955 | | AY540438.1 | |
|  | BeAr162 | | Brazil | | 1955 | | AY540439.1 | |
|  | BeAr189 | | Brazil | | 1955 | | AY540440.1 | |
|  | BeAn23536 | | Brazil | | 1960 | | AY540441.1 | |
|  | BeAr46299 | | Brazil | | 1962 | | AY540442.1 | |
|  | BeAr233436 | | Brazil | | 1973 | | AY540452.1 | |
|  | BeH233393 | | Brazil | | 1973 | | AY540453.1 | |
|  | SPH258595 | | Brazil | | 2004 | | FJ875517.1 | |
|  | SPAn288183 | | Brazil | | 2008 | | FJ875518.1 | |
|  | SPAn289568 | | Brazil | | 2008 | | FJ875520.1 | |
|  | BeH613582 | | Brazil | | 1999 | | HM582843.1 | |
|  | BeH107714 | | Brazil | | 1966 | | AY540444.1 | |
|  | BeAn142028 | | Brazil | | 1968 | | AY540445.1 | |
|  | BeH141816 | | Brazil | | 1968 | | AY540446.1 | |
|  | BeAr142658 | | Brazil | | 1968 | | AY540447.1 | |
|  | BeH203410 | | Brazil | | 1971 | | AY540449.1 | |
|  | BeAr350397 | | Brazil | | 1978 | | AY540454.1 | |
|  | BeH385780 | | Brazil | | 1980 | | AY540455.1 | |
|  | BeH379501 | | Brazil | | 1980 | | AY540456.1 | |
|  | BeH413820 | | Brazil | | 1983 | | AY540457.1 | |
|  | BeH425381 | | Brazil | | 1984 | | AY540458.1 | |
|  | BeAr424083 | | Brazil | | 1984 | | AY540459.1 | |
|  | BeAr511437 | | Brazil | | 1991 | | AY540460.1 | |
|  | BeH511843 | | Brazil | | 1991 | | AY540461.1 | |
|  | BeAn510268 | | Brazil | | 1962 | | AY540462.1 | |
|  | BeAr512943 | | Brazil | | 1992 | | AY540463.1 | |
|  | BeAr513008 | | Brazil | | 1992 | | AY540464.1 | |
|  | BeH512772 | | Brazil | | 1992 | | AY540465.1 | |
|  | BeAr513060 | | Brazil | | 1992 | | AY540466.1 | |
|  | BeAr513292 | | Brazil | | 1992 | | AY540467.1 | |
|  | BeAr527785 | | Brazil | | 1994 | | AY540468.1 | |
|  | BeAr527198 | | Brazil | | 1994 | | AY540469.1 | |
|  | BeAr527547 | | Brazil | | 1994 | | AY540470.1 | |
|  | BeAr544276 | | Brazil | | 1996 | | AY540472.1 | |
|  | Tennessee | | Brazil | | 1996 | | AY540473.1 | |
|  | BeAR378600 | | Brazil | | 1980 | | JF912179.1 | |
|  | BeH394880 | | Brazil | | 1981 | | JF912180.1 | |
|  | BeH413820 | | Brazil | | 1983 | | JF912181.1 | |
|  | BeH422973 | | Brazil | | 1984 | | JF912182.1 | |
|  | BeH423602 | | Brazil | | 1984 | | JF912183.1 | |
|  | BeH463676 | | Brazil | | 1987 | | JF912184.1 | |
|  | BeAR513008 | | Brazil | | 1992 | | JF912185.1 | |
|  | BeH526722 | | Brazil | | 1994 | | JF912186.1 | |
|  | BeH622205 | | Brazil | | 2000 | | JF912187.1 | |
|  | BeH622493 | | Brazil | | 2000 | | JF912188.1 | |
|  | BeAR646536 | | Brazil | | 2001 | | JF912189.1 | |
|  | BeH655417 | | Brazil | | 2002 | | JF912190.1 | |
|  | FMD1240 | | Peru | | 2007 | | HM582844.1 | |
|  | 15094 | | Peru | | 1999 | | HM582846.1 | |
|  | BeAr631464 | | Brazil | | 2001 | | HM582848.1 | |
|  | ARV0548 | | Peru | | 1995 | | AY161946.1 | |
|  | TVP11649 | | Trinidad and Tobago | | 2009 | | HM582840.1 | |
|  | TVP11687 | | Trinidad and Tobago | | 2009 | | HM582841.1 | |
|  | TVP11640 | | Trinidad and Tobago | | 2009 | | HM582842.1 | |
|  | 35720 | | Venezuela | | 1998 | | AY540489.1 | |
|  | 4A | | Venezuela | | 2005 | | KM388821.1 | |
|  | 5A | | Venezuela | | 2005 | | KM388822.1 | |
|  | Cepa1 | | Peru | | 1995 | | AY161939.1 | |
|  | CAREC797984 | | Trinidad and Tobago | | 1979 | | AY540481.1 | |
|  | CAREC889920 | | Trinidad and Tobago | | 1988 | | AY540482.1 | |
|  | P128MC | | Venezuela | | 1959 | | AY540487.1 | |
|  | PHO42H | | Venezuela | | 1961 | | AY540488.1 | |
|  | 35708 | | Venezuela | | 1998 | | AY540490.1 | |
|  | TVP11646 | | Trinidad and Tobago | | 2009 | | HM582839.1 | |
|  | TVP11767 | | Trinidad and Tobago | | 2009 | | HM582851.1 | |
|  | Ecuador79 | | Ecuador | | 1979 | | U52398.1 | |
|  | Panama74 | | Panama | | 1974 | | U52404.1 | |
|  | Trinidad54 | | Trinidad and Tobago | | 1954 | | U52416.1 | |
|  | 1362/77 | | Peru | | 1977 | | AY161927.1 | |
|  | 1368 | | Peru | | 1977 | | AY161928.1 | |
|  | 287/78 | | Peru | | 1978 | | AY161930.1 | |
|  | R35740 | | Peru | | 1979 | | AY161931.1 | |
|  | 1899/81 | | Peru | | 1981 | | AY161932.1 | |
|  | 1914 | | Peru | | 1981 | | AY161933.1 | |
|  | ARVO544 | | Peru | | 1995 | | AY161934.1 | |
|  | OBS2240 | | Peru | | 1995 | | AY161940.1 | |
|  | OBS2250 | | Peru | | 1995 | | AY161941.1 | |
|  | HEB4240 | | Peru | | 1995 | | AY161942.1 | |
|  | HEB4245 | | Peru | | 1995 | | AY161943.1 | |
|  | HEB4246 | | Peru | | 1995 | | AY161944.1 | |
|  | OBS2243 | | Peru | | 1995 | | AY161945.1 | |
|  | OBS6530 | | Peru | | 1998 | | AY161947.1 | |
|  | 3535098 | | Peru | | 1998 | | AY161948.1 | |
|  | OBS6745 | | Peru | | 1998 | | AY161949.1 | |
|  | INS382060 | | Colombia | | 2000 | | AY540474.1 | |
|  | V528A | | Colombia | | 1979 | | AY540475.1 | |
|  | INS347613 | | Colombia | | 1985 | | AY540476.1 | |
|  | 1345 | | Ecuador | | 1981 | | AY540477.1 | |
|  | OBS5041 | | Ecuador | | 1997 | | AY540478.1 | |
|  | CAREC890692 | | Trinidad and Tobago | | 1989 | | AY540483.1 | |
|  | CAREC891954 | | Trinidad and Tobago | | 1989 | | AY540484.1 | |
|  | CAREC891957 | | Trinidad and Tobago | | 1989 | | AY540485.1 | |
|  | 6A | | Venezuela | | 2005 | | KM388814.1 | |
|  | 9A | | Venezuela | | 2007 | | KM388815.1 | |
|  | 10A | | Venezuela | | 2010 | | KM388816.1 | |
|  | 2A | | Venezuela | | 2004 | | KM388817.1 | |
|  | 8 | | Venezuela | | 2006 | | KM388818.1 | |
|  | 3 | | Venezuela | | 2005 | | KM388820.1 | |

YFV: Yellow fever virus. ID: identification. This dataset contained 125 sequences spanning 651 nt [from the nucleotide 644 up to 1,294 of ES504 (KY885000) sequence]. This dataset included 18 sequences of BR-YFV obtained in 2017 and 2018 plus 57 sequences of BR-YFV from previous years (BR-YFV sequences: n=75).
